# Supplementary material for: Data augmentation using Variational Autoencoders for improvement of respiratory disease classification
Source: PLoS One. 2022 Aug 12;17(8):e0266467. doi: 10.1371/journal.pone.0266467 (PMC9374267; doi:10.1371/journal.pone.0266467)
Supplement: Attachment — Submitted filename: VAE Paper Review response.docx [file pone.0266467.s001.docx]

**Original Manuscript ID:** PONE-D-21-27133

**Original Article Title:** Variational Autoencoders for Audio Based Data Augmentation to Enhance Respiratory Disease Classification

**To:** PLOS ONE Editor

**Re:** Response to reviewers

Dear Editor,

Thank you for allowing a resubmission of our manuscript, with an opportunity to address the reviewers’ comments.

We are uploading (a) our point-by-point response to the comments (below) (response to reviewers), (b) an updated manuscript with yellow highlighting indicating changes, and (c)a clean updated manuscript without highlights (PDF main document).

Best regards,

Jane Saldanah,Shaunak Chakraborty,Shruti Patil ,Satish Kumar and Ketan Kotecha

**Reviewer#1, Concern # 1:** The authors need to discuss precisely how each model was run on the dataset, what was learned, what was the data generation strategy from the conditional and unconditional models, how these two models differed in terms of execution.

**Author response:** Thank you for your kind suggestion.

**Author action:** We have added explanation about the training procedure and data generation strategy for the VAE models in section 6.3 (highlighted in yellow).

**Reviewer#1, Concern # 2:** The concept of applying data augmentation strategies to most types of data (including audio) is fairly well explored. But more importantly, a few cited references such as [20] have considered similar directions as the proposed work. I would like to see a more thorough analysis of the knowledge gaps and shortcomings of the earlier papers that are bridged by the current study.

**Author response:** Thank you for your suggestions.

**Author action:** We have added two paragraphs on page number 10 in section 4 / Research Gaps describing the novelties of our work and the issues we noticed in previous papers, such as [30], that have been addressed in this study.

**Reviewer#2, Concern # 1:** Though the work address the problem of imbalanced signal data for respiratory disease diagnosis, the performance analysis is not clear. Data augmentation can only be used for training data. The validation should be done only on original test data.

**Author response:** Thank you for the suggestion.

**Author action:** We have added the details of the train and test sets used for building the VAEs and classification models in section 3 / Exploratory Data Analysis (highlighted in yellow). The system architecture diagram has also been updated to give a clearer picture of the performance evaluation of the classifiers. As per the suggestion received, the synthetic data was used for augmenting the training set only. Hence, we re-evaluated the performance of the classifiers on the hold-out test set, containing real audio samples only. The updated performance metrics, including the confusion matrices, statistical analysis and other graphs have been reported in the manuscript.

**Reviewer#2, Concern # 2:** The similarity of the augmented signal and original signal should be analyzed even more rigorously for classes for which the available data is very much limited, namely for classes.

**Author response:** Thank you for your kind suggestion.

**Author action:** We have further elaborated our contributions in sections 7.1.3 and 7.1.4. We have measured the cross-correlation and Mel cepstral distortion between the real and synthetic samples for all minority classes to elaborate the similarity of the synthetic samples w.r.t the real samples.

**Reviewer#2, Concern # 2:** The data augmentation leads to only a marginal increase in signal classification accuracy. Justify?

**Author response:** Thank you for the suggestion.

**Author action:** On observing the performance metrics of the classifiers aggregated over all classes, it does seem like the augmentation had a little improvement. However, upon comparing the performance metrics for the minority classes with the imbalanced and augmented training sets, a significant difference can be observed. For example, minority classes such as LRTI were being completely misclassified with the imbalanced training set ; post augmentation a significant improvement in the performance metrics can be observed.
